# Child-Parent Interaction Quality Shows Opposite Relationships with Language Comprehension Skill and Autism Symptomatology

**DOI:** 10.1007/s10803-025-07095-1

**Published:** 2025-10-24

**Authors:** Leela Shah, Analia Marzoratti, Tara L. Hofkens, Rose Nevill, Robert C. Pianta, Kevin A. Pelphrey, Anthony J. Krafnick, Tanya M. Evans

**Affiliations:** 1 School of Education and Human Development, University of Virginia, Charlottesville, VA, USA; 2 School of Medicine and Public Health, University of Wisconsin-Madison, Madison, WI, USA; 3 Department of Neurology, University of Virginia, Charlottesville, VA, USA; 4 Department of Psychology, Dominican University, River Forest, IL, USA

**Keywords:** Reading, Comprehension, Autism spectrum disorder, Social behavior, Literacy, Child, Parent

## Abstract

**Purpose:**

Childhood literacy predicts long-term learning outcomes and comprises decoding and linguistic comprehension skills. Decoding involves pattern recognition and sentence parsing, while linguistic comprehension requires understanding semantic context. Each component may differentially relate to social processing, carrying implications for reading among children with autism spectrum disorder (ASD) who often exhibit atypical social processing and communication.

**Methods:**

We investigated relationships between social behavior patterns and literacy subskills among children aged 6–11 with (*n* = 18) and without (*n* = 27) ASD. We examined associations between behavioral attunement during a cooperative task between children and their parent, children’s scores on standardized reading assessments, and children’s autism symptoms. Behavioral attunement was coded through video recordings of child-parent interactions.

**Results:**

Controlling for general intelligence, behavioral attunement was positively associated with reading comprehension and negatively associated with phonemic decoding and autism symptom severity in neurotypical and autistic children. While behavioral attunement’s positive relationship with reading comprehension was driven by the subsample with ASD, its negative relationships with phonemic decoding and autism symptoms were only present for the full sample.

**Conclusion:**

These findings support a significant influence of social processing on linguistic comprehension skills, particularly among children with ASD, as well as an influence of autism symptoms on behavioral attunement, even in children without a formal ASD diagnosis.

## Background

Early literacy is essential for long-term educational and life outcomes (Cunningham & Stanovich, 1997; Farrant & Zubrick, 2012; Lonigan et al., 2000; Sparks et al., 2014). Children’s reading development is partly shaped by their social contexts and experiences. Maternal reading attitudes influence children’s own reading attitudes, even at preliterate ages, and supportive home literacy environments has been linked to language- and literacy-related brain development, independent of income (Altun et al., 2022; Hutton et al., 2020). These socially dependent effects may be especially relevant for children with autism spectrum disorder (ASD), a condition characterized by differences in social communication and restricted and repetitive behaviors (American Psychiatric Association, 2013; Grimm et al., 2018; Lord et al., 2020). This study examines the implications of social processing, captured through behavioral attunement in a naturalistic paradigm, for reading skills in children with and without ASD.

## Integrated Development of Early Literacy and Social Processing Skills

There is evidence for a *bidirectional* relationship between social processing and child literacy (Hutton et al., 2020; Montroy et al., 2014; Niklas et al., 2016; Sparapani et al., 2018). Children’s teacher-reported social skills have been shown to predict reading comprehension and expressive and receptive vocabulary size, primary components of early literacy (Montroy et al., 2014; Sparapani et al., 2018). Conversely, vocabulary and reading skills are thought to play a role in peer social success by providing linguistic tools for improved communication (Sparapani et al., 2018). Social reading experiences, such as joint story reading, can also improve children’s *theory of mind*, the ability to infer the mental states of others (Kidd & Castano, 2013; Westby & Robinson, 2014).

Child-parent relational dynamics influence reading skill development. Shared book-reading with caregivers is often a child’s first exposures to print and supports children’s social, emotional, and academic development (Niklas et al., 2016). Caregivers’ self-reported reading frequency of reading to their young children are also found to predict improved longer-term literacy and cognitive skills (Kalb & van Ours, 2014; Mol & Bus, 2011; Parish-Morris et al., 2013; Zivan & Horowitz-Kraus, 2020). Caregivers’ ability to provide consistent learning environments may underlie these effects, with their reports of shared reading experiences’ frequency, positivity, and reciprocity predicting children’s language literacy outcomes (Aram, 2008, 2012; Bergin, 2001; Lukie et al., 2014; Seror, 2022). More broadly, supportive environments foster children’s positive self-concepts and openness to new learning (Bergin, 2001; Seror, 2022). In sum, both the quality and quantity of caregiver-child interactions likely shape early literacy. Notably, most studies of this reciprocal association rely on self-reported social metrics, whereas the present study instead assesses social behavior during a naturalistic interaction paradigm as an indicator of a child’s applied social processing skills.

## Cognitive Processes Underlying Literacy: The Simple View of Reading

**Fig. 1:**
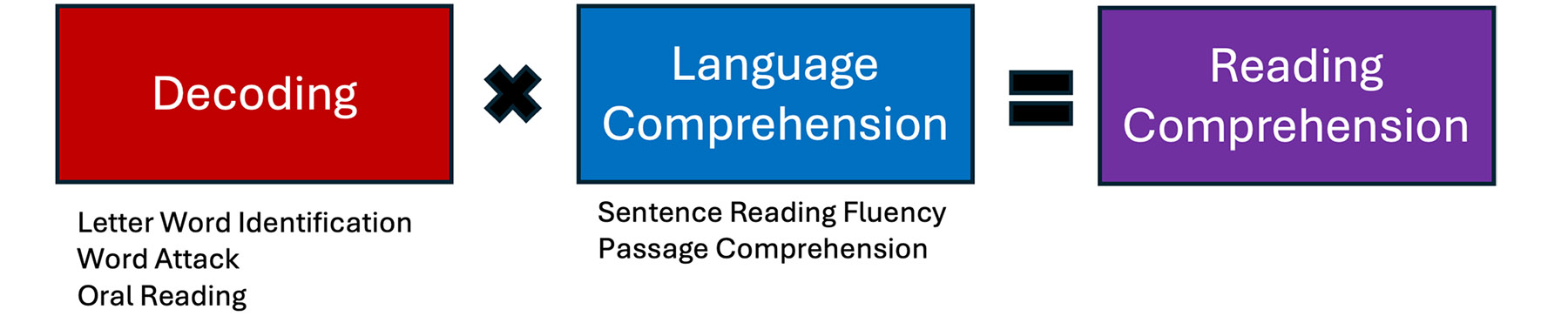
Visual representation of the simple view of reading and corresponding WJ-IV subtests. The model depicted comes from Gough and Tunmer (1986). Below each subcomponent of literacy are listed the subtests of the Woodcock-Johnson (WJ-IV) assessment whose primary skills evaluated correspond with that subcomponent. Task descriptions can be found in Table 1

Reading comprehension is defined by the *Simple View of Reading* (Fig. 1) as the product of *phonemic decoding* (the ability to break down written language using phonics) and language *comprehension* (the ability to understand written text’s semantic meaning) and requires instruction in both skills (Gough & Tunmer, 1986; Juel et al., 1986). The language comprehension component may be especially implicated in the relationship observed between social processing and literacy outcomes. For example, caregivers’ child-directed speech more strongly influences vocabulary and reading when interactions are reciprocal, engaging the child socially (Choi et al., 2021; Jouanjean-L’Antoëne, 1997; Rowe, 2012). Processing social cues (e.g., inferring speaker intention) also supports figurative language comprehension, which requires extracting linguistic meaning by integrating various forms of semantic information (Pexman et al., 2005). Neuroscience further shows that brain areas for Theory of Mind and social cognition interact with language regions during comprehension of social action verbs (Grimm et al., 2018; Lin et al., 2015; Paunov et al., 2019). Decoding, by contrast, shows little interaction with social processing abilities, a distinction well illustrated in research on language skills in autism spectrum disorder (Huemer & Mann, 2010; Vulchanova et al., 2015).

## Autism Characteristics, Social Processing, and Literacy

Autism characteristics are associated with reading comprehension challenges (American Psychiatric Association, 2013; Grimm et al., 2018; Lord et al., 2020) despite often intact decoding skills (Huemer & Mann, 2010). Some autistic children show “hyperlexia”, decoding text early in life without comparable comprehension skills for written or spoken language (Ostrolenk et al., 2017). This suggests that difficulties with reading comprehension may relate to top-down cognitive processes separate from text decoding (Huemer & Mann, 2010; Vulchanova et al., 2015). Supporting this, autistic children often struggle with understanding figurative language (Vulchanova et al., 2015). One explanation is that autism-related differences in social cognitive processes hinder the higher-level integration of contextual and linguistic information necessary for comprehension (Brock et al., 2008; Rundblad & Annaz, 2010).

Further, reading comprehension among autistic children varies as a function of autism characteristics, particularly in social communication domains (McIntyre et al., 2017a, 2017b; Ricketts et al., 2013). These social-cognitive differences, which include difficulties with theory of mind, pragmatic language, and interpreting non-literal meaning, may interfere with the integration of linguistic and contextual information necessary for comprehending written text (Brock et al., 2008; Norbury, 2005; Vulchanova et al., 2015). For instance, autistic children often struggle with understanding figurative or inferential language (Rundblad & Annaz, 2010), a challenge thought to reflect broader differences in social processing and semantic integration.

Neuroimaging research reveals differences in reading-related brain activation linked to top-down semantic processing between autistic and neurotypical children (Alho et al., 2021; Bednarz et al., 2017; Fan et al., 2021). This work implicates difficulties with processing semantic context rather than difficulty decoding text in autism-related reading comprehension challenges (McIntyre et al., 2017a, 2017b). The current study builds on prior work examining semantic and social processing differences in ASD (Brock et al., 2008; Koehne et al., 2016) and extends it by investigating the relationship between autism characteristics, behavioral attunement (a social processing indicator), and reading comprehension across a broader continuum of traits in both autistic and neurotypical children.

## The Current Study

This study examined whether components of reading comprehension differentially relate to child-parent interaction dynamics and continuous autism characteristics in neurotypical and autistic children. We assessed associations between observer-coded child-parent behavioral attunement during a cooperative task, scores reflecting written language comprehension and decoding literacy subskills from the Woodcock-Johnson IV Tests of Achievement (WJ-IV ACH), and autism characteristics among both neurotypical and autistic children aged 6–11, as measured by the Autism Spectrum Quotient - Child version (AQ-Child; Auyeung et al., 2008). Controlling for age and sex, we hypothesized that higher written language comprehension scores would predict greater behavioral attunement, reflecting the social nature of written language comprehension skills, while decoding scores would show no social associations. We further expected behavioral attunement and written language comprehension to negatively correlate with autism characteristics, with decoding unrelated.

## Methods

### Study Recruitment

We recruited 45 parent-child dyads (27 neurotypical [NT], 18 autistic [ASD]) aged 6–11 for two sessions: a phenotypic assessment and a dyadic task. Detailed sample information is in Table 2. Participants were drawn from a racially and socioeconomically diverse database of families within the greater Albemarle County, VA community. The study was approved by the institutional review board (IRB). Parents provided written informed consent. In accordance with the IRB policies for the data collection site, children aged 7 and older provided assent. Families received $100 in gift cards, and children earned small prizes for attending each session.

### Phenotypic Assessment

Children completed the Differential Abilities Scale 2nd edition (DAS-2) and the Woodcock-Johnson IV Tests of Achievement (WJ-IV ACH) during the phenotypic session, administered by a trained psychometrician under clinical supervision (Elliott et al., 2018; Schrank & Wendling, 2018). The DAS-2 assesses cognitive ability, yielding a general conceptual ability (GCA) score (M = 100, SD = 15) with strong reliability (test-retest *r =.79-.94*; Elliott, 1990, 2007). The WJ-IV ACH evaluates academic skills across reading, writing, and math, providing standardized v-scale scores (M = 15, SD = 5). This study focused on WJ-IV reading subtests: Letter-Word Identification, Passage Comprehension, Word Attack (phonetic decoding), Oral Reading, and Sentence Reading Fluency (test-retest *r* =.85-.93; Ding & Alfonso, 2016; Schrank & Wendling, 2018; Villarreal, 2015). See Table 1; Fig. 1 for subtest descriptions and relations to the Simple View of Reading; Table S3 (Supplemental) shows subtest correlations. Parents of both NT and ASD children completed the Autism Spectrum Quotient-Child (AQ-Child), a reliable 50-item questionnaire measuring autism traits in children ages 4–11 across five sub-domains: social skills, communication, attention switching, attention to detail, and imagination (test-retest *r* =.70; Baron-Cohen et al., 1991). Higher scores indicated more autism characteristics. Sample items are in Table S1 (Supplemental material).

### Autism Diagnosis Confirmation

ASD diagnoses were confirmed using the Autism Diagnostic Interview-Revised (ADI-R; Lord et al., 1994), a validated parent interview with strong reliability in autistic children (Lecavalier et al., 2006). Parents completed the ADI-R via Zoom with a research-reliable team member. Inclusion in the ASD participant group required scoring above cutoff on all four ADI-R subscales (social interaction, communication, restricted/repetitive behaviors, developmental differences < 36 months of age). The AQ-Child served as supplementary evidence for diagnosis; autistic individuals typically score above 75 (> 50th percentile). However, participants scoring below this cutoff were still included in the ASD group based on clinical diagnosis.

### Behavioral Task

The experimental task was a cooperative visuospatial tower-building activity lasting approximately three minutes. Participants were presented with a pre-constructed Jenga^®^ tower, then instructed to remove blocks one by one in turns from the tower and to place each removed block on top (for a demonstration of Jenga^®^ gameplay, see www.hasbro.com). Dyads were instructed to play continuously throughout the entire three-minute task, working together to build the tallest tower possible without explicitly mandating turn-taking. Dyad members were seated across from one another at a table and video-recorded to capture nonverbal engagement cues such as eye contact. Children were told they would receive stickers corresponding to the number of levels in their tower following task completion.

### Positive Synchrony Coding

Child-parent behavioral attunement was coded from task videos using an eight-point scale adapted from the *Positive Synchrony Coding Manual* (Criss et al., 2003; see Fig. S1 in the Supplemental material). Higher scores indicated greater eye contact, shared focus, mutual responsiveness, and balanced leadership, while lower scores reflected ignoring, interruptions, or unequal dominance. Coders were given anchor points defining each score, from 1 (parallel activity with no interaction) to 8 (visible reciprocal engagement). All sessions were independently coded by two trained raters, achieving high inter-rater reliability (ICC = 0.93). The final synchrony score was the average of both raters.

### Primary Statistical Analyses

We first examined how AQ-Child scores and literacy subskills predicted child-parent behavioral attunement during interaction, assessing whether written language comprehension and decoding differentially relate to social ability. Analyses were conducted in R 4.2.2 using the “stats” package. Descriptive statistics and correlations are reported in Table 2 and Table S2 (Supplemental material). Age was excluded as a covariate as it was not correlated with other variables and WJ-IV ACH scores were age-standardized (Bulut et al., 2021). Baseline group differences in WJ-IV ACH subtests were assessed via independent samples t-tests (Table S2, Supplemental material). Covariates in all estimated models included child sex (male = 0) and child IQ (standardized DAS-II conceptual ability scores). Continuous variables were standardized prior to modeling.

We then ran regression models predicting behavioral attunement from each WJ-IV ACH subtest separately (Table S4, Supplemental material), followed by a multivariable model including multiple subtests and AQ-Child scores (Table 3). Variance Inflation Factors (VIF) were checked for multicollinearity, with a cutoff of VIF < 10 based on prior work testing multiple WJ-IV ACH scores in the same model (Locke, 2011; Speece et al., 2011). Reading subtests exceeding this threshold were excluded: Oral Reading (VIF = 11.31) and Letter-Word Identification (VIF = 11.17). Remaining subtests’ scores had VIF < 3. Models were compared for relative fit by assessing (1) adjusted R^2^ and (2) second-order AIC (AICc), adjusted for small samples (Portet, 2020). P-values were adjusted for false discovery rate using Benjamini-Hochberg correction (q-values) via the *p.adjust* function (Benjamini & Hochberg, 1995; Jafari & Ansari-Pour, 2019). Residuals met normality assumptions based on Shapiro-Wilk test (*p* >.5), Q-Q plots, and density curves. Statistical power was assessed using the pwr package and standardized betas as effect sizes (Champely et al., 2020).

### Moderation Analysis

To evaluate whether autism characteristics moderates the relationship between behavioral child-parent attunement and literacy subskills, we ran separate regressions predicting scores for WJ-IV subtests significantly related to attunement in the full model. Each model predicted the reading subtest score based on AQ-Child, behavioral attunement, and their interaction (Table S6a-b, Supplemental material).

### Robustness Analyses

We re-estimated the full model and significant interaction models using a binary ASD diagnosis variable instead of the continuous AQ-Child scores to determine whether a clinical autism diagnosis moderates the relationship between behavioral attunement and literacy skills, beyond what is explained by autism characteristics alone (Table S5, Table S6c-d, Supplemental material).

To evaluate whether results were driven by either subgroup, we conducted leave-one-out cross-validation (LOO-CV) of the multivariable regressions from our primary analysis separately for ASD (*n* = 16), NT (*n* = 29), and the full sample (Wong, 2015; Yates et al., 2023). This approach ensured robust findings within smaller subgroups and allowed direct comparison to the full sample (see Table S7, Supplemental material).

## Results

There was a higher proportion of males in the ASD group than in the NT group (87.5% vs. 58.6%, *p* =.03). Mean ages did not differ significantly for children (ASD: 8.28 [1.57] years; NT: 9.20 [1.84] years) or parents (ASD: 38.3 [4.8] years; NT: 41.5 [5.9] years). Children with ASD had significantly higher AQ scores (ASD: 107.4 [18.8]; NT: 48.0 [20.6], *p* <.01) and lower IQ (ASD: 101.4 [18.0]; NT: 116.6 [13.3], *p* =.01). Significant group differences were also found in Oral Reading (ASD: 94.8 [22.6]; NT: 109.1 [18.2], *p* =.04) and Word Attack (ASD: 96.8 [19.9]; NT: 113.5 [17.2], *p* =.01) but not Sentence Reading Fluency. Behavioral attunement was significantly lower in the ASD group (4.72 [1.20] vs. 5.90 [1.22], *p* <.01). See Table 2 for full subgroup comparisons.

### Predictors of Child-Parent Behavioral Attunement

Controlling for sex and IQ, the full model showed that children’s literacy subskills and autism characteristics predicted child-parent behavioral attunement (F (6, 38) = 4.09, *p* =.003, Adjusted R^2^ = 0.30, AIC_c_ = 124.28, Table 3). Behavioral attunement was positively associated with Sentence Reading Fluency (β = 0.47, *p* =.026, VIF = 2.56), a written language comprehension measure, and negatively associated with AQ-Child scores (β=−0.41, *p* =.008, VIF = 1.33) and scores in Word Attack (β=−0.35, *p* =.042, VIF = 1.76), a phonemic decoding measure. These patterns held in analyses using binary ASD diagnosis and LOO-CV on the full sample (Tables S5, S7). Although these associations did not survive multiple comparison correction (q-values 0.07–0.09), effect sizes suggest meaningful relationships: a 1 SD increase in Sentence Reading Fluency or Word Attack corresponded to 47% and − 35% SD changes in attunement, respectively.

### Interactions Between ASD and Behavioral Attunement Scores

**Fig. 2:**
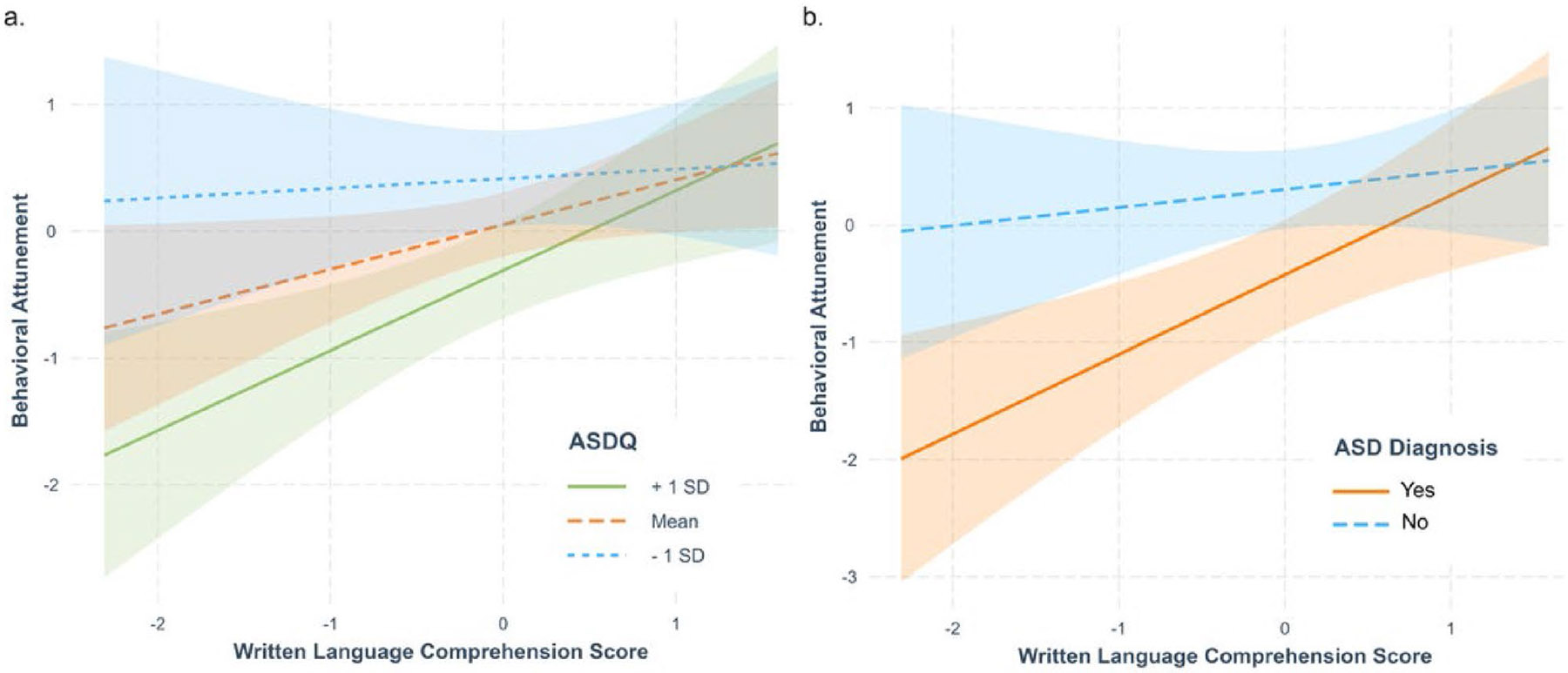
Interaction between autism symptomatology and written language comprehension in their associations with behavioral attunement. Interaction plots depict the differential relationships between behavioral attunement scores and written language comprehension skill as reflected by scores on the Woodcock-Johnson (WJ-IV) assessment sentence reading fluency subtest (a) across continuous levels of ASD characteristics measured via ASDQ and (b) based on diagnosis of ASD. Covariates include sex and IQ

A significant interaction emerged between Sentence Reading Fluency (β = 0.35, *p* =.04, VIF = 1.74) and AQ-Child scores (β= −0.36, *p* =.01, VIF = 1.26), indicating that more autism characteristics correlated with stronger positive association between written language comprehension and behavioral attunement (Sentence Reading Fluency*AQ-Child: β = 0.28, *p* =.03, VIF = 1.05; Fig. 2a; Table S6a-b, Supplemental material). This was supported by LOO-CV for the full sample (Table S7). A similar interaction was found with binary ASD diagnosis (Fig. 2b). No interaction was observed between AQ-Child and Word Attack scores.

### Robustness Analysis of Participant Subgroups Based on ASD Diagnosis

To examine whether autism heterogeneity influenced results and if effects were driven by autistic participants, we used leave-one-out cross-validation (LOO-CV) to estimate models separately for the ASD (*n* = 16), NT (*n* = 29), and full samples (see Table S7, Supplemental material). Potential shortcomings of this approach are addressed in the limitations.

**Fig. 3:**
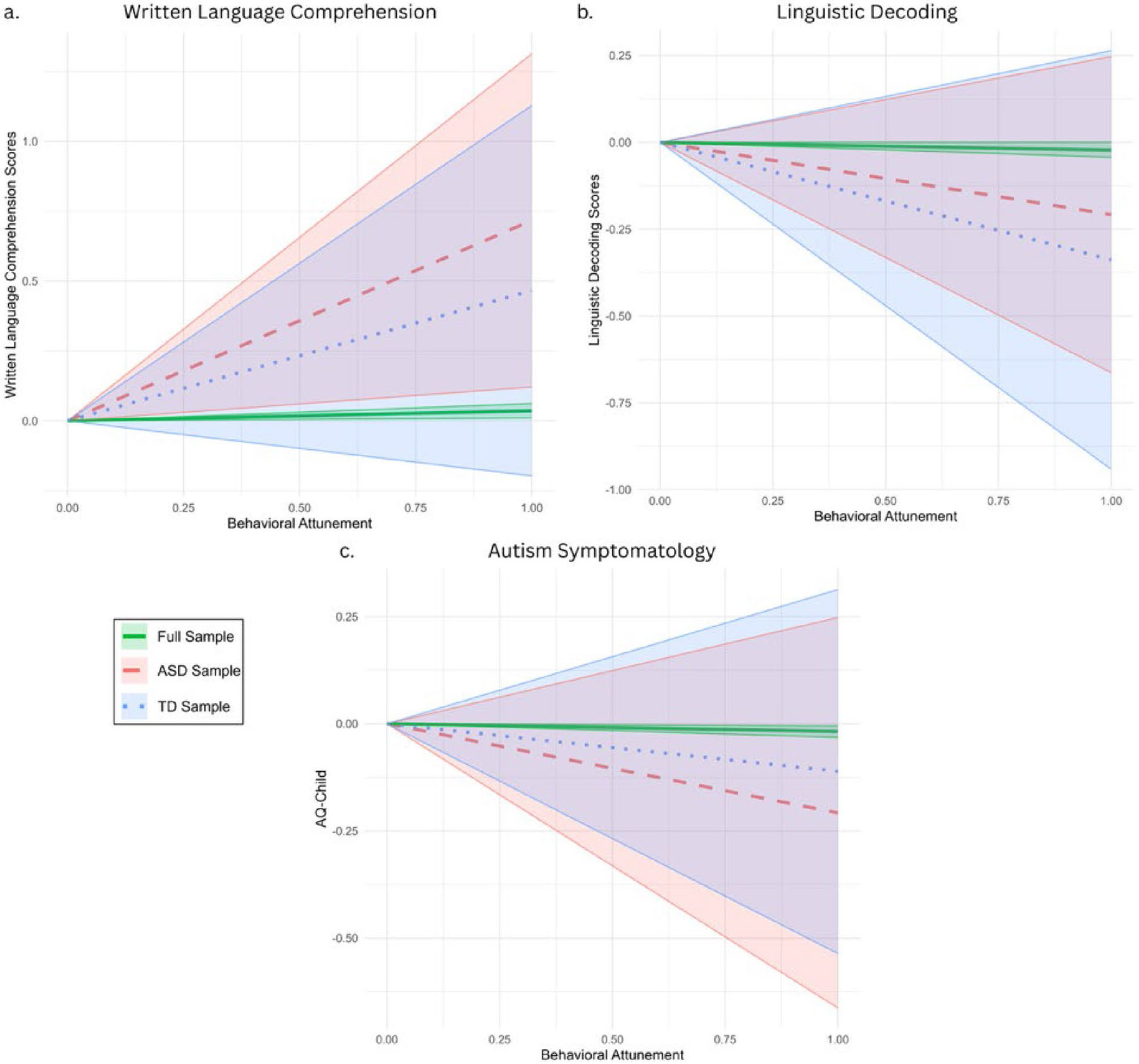
Positive association between written language comprehension and behavioral attunement is driven by the autistic subsample. Results from leave-one-out cross-validation for autistic (*N* = 18) and typically developing (*N* = 27) subsamples as well as the full sample (*N* = 45). Results reflect full models for a given sample predicting behavioral attunement based on scores reflecting (a) written language comprehension and (b) decoding literacy subskills, and (c) autism spectrum quotient (ASDQ). Covariates include sex and IQ. The positive relationship seen for comprehension scores emerged in the ASD subsample, while all other effects emerged in the full sample only. See detailed procedure in methods

In the ASD group, written language comprehension was positively associated with behavioral attunement in the main-effects only model (β = 0.72, *p* =.03; Fig. 3a). This association was not significant in NT children. Negative relationships between decoding and attunement, and between AQ-Child and attunement, emerged only in the full sample (decoding: β = −0.02, *p* =.045; AQ-Child: β = −0.02, *p* =.01), but not within ASD or NT subgroups, indicating these effects reflect between-group differences rather than within-group variability. Large confidence intervals for decoding associations within subsamples further confirmed non-significance (Fig. 3b). Similarly, only the full sample showed a significant interaction between AQ-Child and WJ written language comprehension scores (β = 6.7 × 10^−4^, *p* =.04).

## Discussion

Early social ability and reading comprehension are closely and bidirectionally linked (Hutton et al., 2020; Montroy et al., 2014; Sparapani et al., 2018). Autistic individuals often face reading challenges, thought to be rooted in linguistic and social comprehension difficulties (Grimm et al., 2018; Ricketts et al., 2013; Vulchanova et al., 2015). Our findings extend this evidence by showing that reading comprehension components differ in their associations with social interaction skills, moderated by autistic traits. Specifically, ability with comprehension of written text’s semantic meaning correlated with behavioral attunement only in autistic children, while ability with decoding text was negatively associated with attunement across the full sample. ASD diagnosis and AQ-Child scores were also negatively related to behavioral attunement, consistent with our hypotheses. Further, our robustness analysis suggested that the associations of behavioral attunement with decoding and AQ-Child, and their interaction with written language comprehension, are mainly driven by autism characteristic differences across groups. The positive association between attunement and written language comprehension is instead primarily driven by the autistic subsample.

The positive correlation between written language comprehension and behavioral attunement in autistic children supports differences in the cognitive demands of literacy components, especially regarding their links to social processing (Parkin, 2021; Schrank & Wendling, 2018; Villarreal, 2015). While comprehension, a higher-level literacy skill, likely engages neurocognitive processes involved in social cognition, decoding, a lower-level skill, does not. This association’s emergence only in autistic children suggests written language comprehension may be a key area of differentiation in this population’s literacy development. Thus, the connection between written language comprehension and child-parent interaction quality implies that social interaction skills captured by our attunement measure may contribute substantially to reading difficulties in autism.

Although our statistical models positioned attunement as the outcome variable for consistency across analyses, these results should not be interpreted as directional. It is equally plausible that children’s social behaviors differentially predicted their literacy skills, or that literacy components differentially support social engagement. Our analyses were correlational and should be interpreted as evidence of a reciprocal link between social interaction quality and reading skills, consistent with other empirical accounts of literacy development (Hutton et al., 2020; Montroy et al., 2014; Niklas et al., 2016; Sparapani et al., 2018).

### Positive Association Between Written Language Comprehension and Behavioral Attunement

Written language comprehension was positively associated with behavioral attunement, but this relationship was significant only within the autistic subgroup (Fig. 3a). This may reflect greater variability in social behavior among autistic children, making attunement more sensitive to differences in written language comprehension. Previous studies indicate that children with strong language comprehension (e.g., understanding stories or connecting language input to prior experiences) tend to display better social processing and interpersonal skills (Montroy et al., 2014; Sparapani et al., 2018). Language comprehension also correlates with theory of mind and mentalization, which enhance integration of social and textual information and support metacognitive reading strategies (Boerma et al., 2017; Li & Yu, 2015; Lin et al., 2024; Nicholson, 1997). Thus, better social skills may contribute to higher reading comprehension, as seen here. The lack of significance in neurotypical children may reflect a ceiling effect in their social interaction quality, limiting variability required for an association to emerge.

As the relationship between social and written language comprehension skills is likely bidirectional, an alternative may be that children with stronger written language comprehension tend to develop improved social skills through increased engagement with texts, often involving integrating social and linguistic content (Arnold et al., 2012; Kidd & Castano, 2013; Trzesniewski et al., 2006; Westby & Robinson, 2014). For example, comprehension of emotion words has been shown to predict scores on empathy assessments in children (Li & Yu, 2015), and reading fiction has been found to predict stronger empathy and social performance (Bal & Veltkamp, 2013; Tamir et al., 2016). The latter interpretation may be especially relevant for autistic children, who often develop language skills less through spontaneous interaction (Diehl et al., 2006; Duffy & Healy, 2011) and more through structured contexts like reading or listening to stories (Kuo et al., 2013; Shane & Albert, 2008). Intervention research supports the benefits of such approaches, with social stories improving socialization in autistic children (Golzari et al., 2015; Mathée-Scott & Ellis Weismer, 2022; Qi et al., 2018; Walton & Ingersoll, 2013). Although further longitudinal studies are needed to clarify causality, our findings highlight a unique correlation between social skills and written language comprehension in autism.

### Negative Association Between AQ-Child or ASD Diagnosis and Behavioral Attunement

As expected, more autism characteristics co-occurred with lower behavioral attunement. This negative correlation between AQ-Child and attunement appeared in the full sample but not within the autistic or neurotypical subgroups (Fig. 3c), suggesting the effect was driven by ASD diagnosis rather than continuous traits. Our moderation analysis showed autistic children had a stronger positive link between reading comprehension and attunement than neurotypical peers, despite similar Sentence Reading Fluency scores-ruling out ceiling effects.

One explanation is that autistic children with more typical social behaviors better apply social processing skills to reading comprehension. Prior research links more socially facilitative interaction behaviors among both TD and ASD children to theory of mind (Chiu et al., 2023; Watson et al., 1999), a skill closely linked to reading comprehension abilities (Atkinson et al., 2017; Dore et al., 2018). Alternatively, higher attunement might reflect closer child-parent interactions that especially benefit autistic children’s social processing and written language comprehension. Child-parent relationship quality has been found to predict children’s social information processing skills, in turn moderating parental influences on children’s learning outcomes (Arbel et al., 2021; Rah & Parke, 2008). Consequently, autistic children, who face unique challenges with social information processing, might exhibit more pronounced differences in written language comprehension based on the level of support provided by their parental relationships.

Future studies could verify this interpretation by assessing how longitudinal parent-child relationship quality impacts social processing, measured through behavioral attunement or other indicators, and reading outcomes in autism (Arbel et al., 2021).

### Negative Association Between Phonemic Decoding and Behavioral Attunement

In contrast to written language comprehension skill, decoding skills were negatively associated with child-parent behavioral attunement during a cooperative task, suggesting that cognitive traits supporting proficient decoding may coincide with reduced social cognition. This effect persisted across the full sample but was not significant within autistic or neurotypical subgroups (Fig. 3b), indicating it was not driven by ASD diagnosis. This aligns with research showing that cognitively demanding tasks can bias social information processing by limiting integration of new social cues and reducing adjustments to prior expectations (Doherty et al., 2017; Stangor & Duan, 1991; Allen et al., 2009; Harris & Perkins, 1995). Such cognitive distraction has even been used to alleviate social anxiety and stress (Blackie & Kocovski, 2016; Janson & Rohleder, 2017). Taken together, this literature suggests that phonemic decoding may draw on cognitive resources distinct from those required for social processing, thus hindering concurrent social engagement.

The autism literature also provides context for this interpretation of the negative relationship observed between decoding skills and child-parent interaction quality. Autistic individuals, who frequently face challenges with social processing, often demonstrate strong phonemic decoding abilities despite difficulties with reading comprehension and semantic processing (Huemer & Mann, 2010; Ostrolenk et al., 2017). However, although decoding was correlated with autism characteristics, the interaction was nonsignificant. Furthermore, autistic children exhibited significantly lower decoding scores compared to NT children. Together, these findings suggest that social skills, rather than autism-specific social characteristics, could explain the negative link between decoding and behavioral attunement.

### Limitations and Future Directions

A key limitation of this study is the modest sample size (27 neurotypical and 18 autistic dyads), below the often-cited threshold of 30 per group and potentially reducing statistical power (Golds et al., 2022). Nonetheless, power analyses revealed substantial effect sizes for the behavioral attunement scale: a 1 SD increase in Sentence Reading Fluency corresponded to a + 47% change in attunement, while a 1 SD increase in Word Attack corresponded to −35%. Given attunement scores range from 1 to 8, these effects may be practically meaningful despite limited statistical significance (Schäfer & Schwarz, 2019; Sullivan & Feinn, 2012). These findings should be validated in larger samples (Bakker et al., 2019).

Several sample characteristics introduce limitations. Children’s average IQ (M = 111.16, SD = 16.65) was higher than the population average, which may affect generalizability (Garcini et al., 2022; Hoard et al., 1999). The sample was composed primarily of mother-child dyads (*N* = 41), with relatively few father-child dyads (*N* = 4), which may limit conclusions across parent-child dynamics (Arbel et al., 2021). This study included more male children (*N* = 31) than female children (*N* = 14), which constrains generalizability of its findings to females with ASD as social dynamics often vary between genders in ASD (Dean et al., 2017). Finally, ASD diagnosis was confirmed with parent report rather than gold-standard diagnostic evaluation, which could introduce bias (Fulceri et al., 2025; Möricke et al., 2016).

While LOO-CV in small samples may result in less stable estimates, we chose it for its established use in behavioral neuroscience (de Rooij & Weeda, 2020; Scheinost et al., 2023), its efficiency with simple models (≤ 5 predictors; Yates et al., 2023), and its advantage of maximizing data use while minimizing over-fitting (Wong, 2015). LOO-CV also allowed consistent comparisons across subgroups of different sizes using a uniform framework, and its slight pessimistic bias likely produced conservative estimates (Geroldinger et al., 2023). Still, results should be interpreted cautiously, with larger samples prioritized in future work.

An additional limitation is the indirect nature of our social processing measure, as behavioral attunement serves as an observed indicator rather than a direct assessment of underlying social processing skills. Behavioral attunement measure was assessed during a cooperative tower-building task, capturing interactive skills but not reading. It was also based on neurotypical social behavior norms that may not fully capture autistic social processes (Criss et al., 2003). Self-reports or individually normed social behavior coding could improve future measurement. Behavioral attunement scores may also reflect both child and parent interactional contributions, as parents, particularly of autistic children, often adapt their behaviors to their child’s social abilities (Bontinck et al., 2018; Rozenblatt-Perkal & Zaidman-Zait, 2020). Further work should clarify relative child versus parent influences on behavioral attunement during social interactions, examine it in broader contexts (e.g., between children and their peers or teachers), and validate it with other indicators of social processing.

Overall, our results underscore the developmental importance of caregiver-child interactions, particularly for autistic children. Policies that promote parent training to this end could strengthen children’s social communication skills as well as their academic outcomes (Ansari & Gershoff, 2016; Roberts et al., 2019). Future research should examine how social attunement frameworks can be embedded in both family-centered interventions and classroom practices to foster more inclusive and effective learning environments.

## Conclusion

This work interrogated relationships between cooperative child-parent social behavior, components of literacy, and autistic traits. Our findings suggest that written language comprehension among autistic children may be positively related to skills contributing to social interaction quality, while text decoding is negatively associated with these social skills across children regardless of their autism diagnosis status. These findings align with prior neural evidence for distinct mechanisms driving these processes, demonstrating unique roles for social cognition roles across critical early reading subprocesses. They also highlight the social nature of literacy development, supporting the role of interventions rooted in social interaction in strengthening reading skills (Vaughn et al., 2015; Young et al., 2015).

## Supplementary Material

Supplement

**Supplementary Information** The online version contains supplementary material available at https://doi.org/10.1007/s10803-025-07095-1.

## Figures and Tables

**Table 1:** Descriptions of Woodcock-Johnson IV (WJ-ACH) subtests measuring reading skills

Subtest	Description	Skills Measured
Letter Word Identification	Initial items require a participant to identify individual letters in bold print. For remaining items (the majority), they are asked to read words of increasing difficulty presented in isolation, not in context.	Recognizing and/or decoding, then pronouncing words.
Word Attack	Initial items require the participant to produce sounds associated with single letters. Remaining items (the majority) require a participant to pronounce non-words of increasing complexity.	Applying phonemic processing and decoding skills to unfamiliar words.
Oral Reading	The participant is required to read aloud sentence of increasing complexity, and assessed for prosody, fluency of expression, and accuracy.	Phonemic decoding of written language. Language comprehension may be involved but is not assessed.
Sentence Reading Fluency	The participant is given a 3-minute time limit to read aloud as many out of a series of simple sentences as they can within this window. As they read, they must circle a response indicating whether the sentences are true or false.	Ability to efficiently read, interpret, and comprehend simple written sentences. While some decoding is involved, the primary skill assessed is written language comprehension.
Passage Comprehension	Participants are presented a short phrase and must match it with the appropriate picture out of three choices. Remaining items (the majority) require the participant to supply a missing word to complete increasingly complex sentences then paragraphs.	Understanding of the semantic meaning underlying written text. Deriving meaning from context by integrating information. While some decoding is involved, the primary skill assessed is written language comprehension.

**Table 2:** Participant demographics and subtest scores

Variable	Full sample		NT		ASD		Odds ratio	95% CI	*p*
	Freq	%	Freq	%	Freq	%			
Female (Child)	14	31.1%	12	41.3%	2	12.5%	0.31	[0.03, 1.66]	.189
Male (Child)	31	68.9%	17	58.6%	14	87.5%	1.28	[0.43, 3.70]	.632
Caucasian/White non-Hispanic	35	77.8%	24	82.8%	11	68.8%	0.83	[0.29, 2.34]	.813
African American/Black	2	4.4%	0	0%	2	12.5%	Inf	[0.66, Inf]	.056
Asian/Asian American	4	8.9%	3	10.3%	1	6.3%	0.61	[0.01, 8.34]	1.00
Hispanic/Latino	6	13.3%	5	17.2%	1	6.3%	0.37	[0.01, 3.73]	.650
Native American/American Indian	1	2.2%	1	3.4%	0	0%	0	[0.00, 73.04]	1.00
Mixed	3	6.7%	3	10.3%	0	0%	Inf	[0.00, 4.86]	.54
Decline to Answer	1	2.2%	1	3.4%	0	0%	0	[0.00, 73.04]	1.00

Variable	Full Sample		NT		ASD		*t*	*df*	*p*	*d*
	M (SD)	Range	M (SD)	Range	M (SD)	Range				
Child age (in years)	8.88 (1.79)	6–11.9.9	9.2 (1.84)	6–11.9.9	8.28 (1.57)	6–11	1.77	35.49	.085	0.53
Parent age (in years)	49.53 (5.76)	31–50	41.46 (5.89)	34–50	38.30 (4.75)	31–49	1.29	36.17	.207	0.38
**AQ-child score** ^a^	**62.22 (28.19)**	**22–112**	**47.96 (20.57)**	**22–111**	**107.43 (18.82)**	**64–112**	**−8.81**	**41.46**	< **.001**	**−2.44**
**AQ score (parent)** ^a^	**14.30 (9.30)**	**0–42**	**12.79 (6.89)**	**0–34**	**17.20 (12.22)**	**0–42**	**−2.34**	**21.30**	**.029**	**−0.84**
**IQ score**	**111.16 (16.65)**	**62–142**	**116.55 (13.33)**	**93–142**	**101.38 (17.98)**	**62–138**	**2.96**	**24.28**	**.007**	**1.00**
Passage comprehension (C)^^^	100.2 (17.84)	40–129	102.69 (17.66)	40–129	95.69 (17.82)	67–123	1.27	30.80	.215	0.40
Sentence reading fluency (C)^^^	101.42 (19.23)	57–132	104.03 (16.35)	60–132	96.69 (23.43)	57–128	1.11	23.24	.277	0.38
**Oral reading (D)** ^^^	**104.02 (20.8)**	**40–135**	**109.1 (18.22)**	**40–135**	**94.81 (22.55)**	**49–126**	**2.17**	**25.95**	**.039**	**0.72**
**Word attack (D)** ^^^	**107.56 (19.71)**	**41–142**	**113.48 (17.23)**	**62–142**	**96.81 (19.85)**	**41–120**	**2.82**	**27.52**	**.009**	**0.92**
Letter word identification (D)^^^	103.31 (17.7)	40–133	106.55 (16.41)	40–133	97.44 (18.94)	50–123	1.62	27.47	.117	0.53
Behavioral attunement	5.48 (1.33)	2–8	5.9 (1.22)	2–8	4.72 (1.2)	2–6	**3.14**	**31.58**	**.004**	**0.97**

We compared neurotypical (NT) and autism spectrum disorder (ASD) diagnosed participant subgroups using Fisher’s Exact Test for categorical variables and T-tests for continuous. Odds ratios were infinite (Inf) in cases with a zero count in one category. Rows containing variables showing significant between-group differences are bolded. Statistics reported for Fisher’s Exact Test include odds ratios their 95% confidence intervals, and p-values, and for T-tests include t-statistics, degrees of freedom (df), p-values, and Cohen’s d as a measure of effect size. Reading subtests labeled “C” are linked with written language comprehension, and those labeled with “D” are linked with decoding

*N*_dyads_ = 45. Data are shown as mean ± SD or percentages. We reported statistics for sex assigned at birth only and using “Male” and “Female” labels alone because all responses to the question fell within these two categories, and were identical to responses for a separate item assessing gender. Statistics describing race/ethnic makeup of the sample were derived from self-reported responses. Participants were able to select multiple categories based on how they and/or their children self-identified (i.e., categories were not mutually exclusive)

^ Predictors derived from WJ-IV standardized test scores

a “AQ” refers to total scores based on parent-reports for the Autism Spectrum Quotient: original version for adults (maximum score = 50, threshold score = 26; Baron-Cohen et al., 1991) and child version for children (maximum score = 150, threshold score = 76; Auyeung et al., 2008)

**Table 3:** Full model: multivariable regression analysis for multiple WJ-IV subtest scores

Outcome: Child-Parent Behavioral Attunement					
Variable	β (SE)	*t*	*p*, *q*	VIF	Power
Intercept	−0.16 (0.16)	−1.05	0.30, 0.42	-	-
Passage Comprehension (C)	0.13 (0.21)	0.61	0.54, 0.63	2.66	0.09
**Sentence Reading Fluency (C)**	**0.47 (0.20)**	**2.32**	0.03*, **0.09**	**2.56**	**0.81**
**Word Attack (D)**	**−0.35 (0.17)**	**−2.10**	0.04*, **0.09**	**1.76**	**0.52**
**AQ-Child**	**−0.41 (0.15)**	**−2.80**	0.01*,**0.07**	**1.33**	**0.67**
Sex	0.53 (0.30)	1.74	0.09, 0.16	1.27	0.26
IQ	−0.08 (0.18)	−0.44	0.66, 0.66	1.93	0.07

Adjusted R^2^ values, standardized beta values (β) with standard errors (SE), t-values, variance inflation factors (VIF), second-order Akaike’s information criterion (AICc), and power estimates are reported. Bolded rows reflect a statistically significant relationship. Both p and q values, which correct for false discovery rate, are reported. Reading subtests labelled ‘C” are associated with written language comprehension, and those labeled with “D” are associated with decoding

* *p* <.05; *q* < 0.05. R^2^ = 0.30; AIC_c_ = 124.28; F (6, 38) = 4.09; Model *p* = .003; Values reported come from regressions which included scores from a single WJ-II subtest and covariates as predictors. Continuous autism spectrum quotient (AQ-Child) was used as a measure of autistic characteristics

## Data Availability

The data analyzed for this study are available upon request from the corresponding author, as participants did not consent for their data to be shared publicly.
