# Supplementary material for: Child-Parent Interaction Quality Shows Opposite Relationships with Language Comprehension Skill and Autism Symptomatology
Source: J Autism Dev Disord. Author manuscript; Available in PMC 2026 Jun 15. (PMC13267008; doi:10.1007/s10803-025-07095-1)
Supplement: Supplement [file NIHMS2176583-supplement-Supplement.docx]

| Table S1. Sample Items from Autism Spectrum Questionnaire – Child Version (AQ-Child, Auyeung et al., 2008) | | |
| --- | --- | --- |
| **Subdomain** | **Representative Items** | **Reverse-coded?** |
| Communication | S/he has difficulty understanding rules for polite behavior. | No |
|  | S/he finds it easy to “read between the lines”  when someone is talking to her/him. | Yes |
| Social skills | S/he prefers to do things with others rather than  on her/his own. | No |
|  | S/he finds it easy to work out what someone is  thinking or feeling just by looking at their face. | Yes |
| Attention switching | New situations make him/her anxious. | No |
|  | S/he finds it easy to go back and forth between  different activities. | Yes |
| Attention to detail | S/he usually notices house numbers or similar  strings of information. | No |
|  | S/he doesn’t usually notice small changes in a  situation, or a person’s appearance. | Yes |
| Imagination | S/he finds it difficult to imagine what it would  be like to be someone else. | No |
|  | S/he finds it very to easy to play games with  children that involve pretending. | Yes |
| Response scale treated as a 4-point Likert scale, with responses scoring 0-3 as marked. Responses included “definitely agree”, “slightly agree”, “slightly disagree”, and “definitely disagree”. The total AQ score consists of the sum all items. Minimum score = 0; Maximum score = 150. See Auyeung et al., 2008 for more detail. | | |

Figure S1. Behavioral synchrony scale.


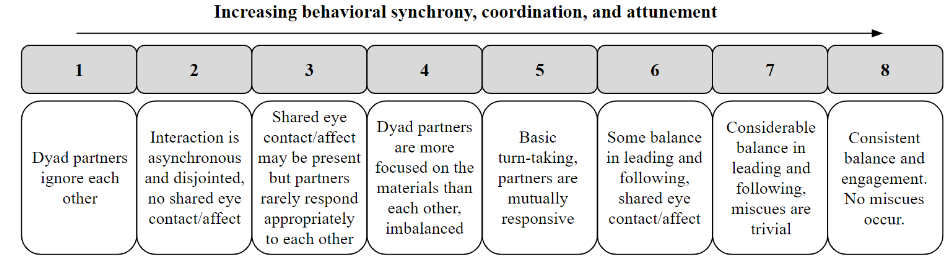


Above are anchor points each score on our behavioral coding scale based on Criss et al. (2003), which was used to code for behavioral attunement in post-hoc viewings of videos of child-parent dyads performing the math task. Each dyad is awarded a score based on social reciprocity, balance, and exhibited affect. While scores range from 1-9 in the original scale, the maximum score was adjusted to 8 points in the current study, as we deemed the criteria of scores 7 and 8 to be too similar to reliably distinguish in our study population. Two raters coded each video separately, and inter-rater reliability, assessed via intraclass correlation coefficient, was 0.93 for this task. The final score consisted of the average of the two raters’ scores.

| Table S2. Correlations among study variables. | | | | | | | | |
| --- | --- | --- | --- | --- | --- | --- | --- | --- |
|  | (1) | (2) | (3) | (4) | (5) | (6) | (7) | (8) |
| (1) Sex | -- |  |  |  |  |  |  |  |
| (2) Age | 0.24  (0.09) | -- |  |  |  |  |  |  |
| (3) AQ-Child | -0.30  (0.09) | -0.22  (0.09) | -- |  |  |  |  |  |
| (4) Parent AQ | 0.00  (0.09) | 0.02  (.915) | **0.59***  **(0.07)** | -- |  |  |  |  |
| (5) Behavioral Attunement | 0.25  (0.09) | 0.00  (0.09) | **-0.46***  **(0.08)** | **-0.34***  **(0.09)** | -- |  |  |  |
| (6) IQ | 0.06  (0.09) | 0.23  (0.09) | **-0.32***  **(0.09)** | -0.26  (0.09) | 0.26  (0.09) | -- |  |  |
| (7) Passage Comprehension Score (C) | -0.11  (0.09) | 0.02  (0.09) | -0.16  (0.09) | -0.20  (0.09) | 0.25*  (0.09) | **0.64***  **(0.07)** | **--** |  |
| (8) Word Attack Score (D) | -0.02  (0.09) | 0.07  (0.09) | -0.25  (0.09) | -0.17  (0.09) | 0.08  (0.09) | **0.47***  **(0.08)** | **0.64***  **(0.07)** | **--** |
| (9) Sentence Reading Fluency Score (C) | -0.19  (0.09) | -0.01  (0.09) | -0.22  (0.09) | **-0.40***  **(0.09)** | 0.36  (0.08) | **0.63***  **(0.07)** | **0.72***  **(0.06)** | **0.63***  **(0.07)** |
| * Correlation is significant at the 0.05 level (one-tailed). Pearson’s correlation coefficients are reported over standard errors. Significant correlations are in bold. Among WJ-II subtest names, ‘C’ refers to those related to linguistic comprehension and ‘D’ refers to those related to decoding. | | | | | | | | |

| Table S3. Paired t-tests comparing scores between WJ-IV ACH subtests. | | | | |
| --- | --- | --- | --- | --- |
|  | Passage Comprehension  (C) | Sentence Reading Fluency  (C) | Word Attack  (D) | Letter Word Identification  (D) |
| Oral  Reading  (C) | ***t* = 3.29**  ***P* = .003***  **M = 7.39**  **(2.77, 12.00)** | t = 1.98  P = .059  M = 3.93  (-0.15, 8.00) | **t = -2.45**  **P = .021***  **M = -4.00**  **(-2.37, -0.93)** | t = 1.84  P = .078  M = 2.67  (-0.32, 5.65) |
| Passage Comprehension  (C) |  | t = -1.26  p = .219  M = -3.46  (-9.12, 2.19) | **t = -4.25**  **p < .001***  **M = -11.39**  **(-16.89, -5.88)** | **t = -2.36**  **p = .03***  **M = -4.72**  **(-8.83, -0.61)** |
| Sentence Reading Fluency  (C) |  |  | **t = -3.43**  **p = .002***  **M = -7.93**  **(-12.67, -3.18)** | t = -0.58  p = .057  M = -1.26  (-5.74, 3.22) |
| Word  Attack  (D) |  |  |  | **t = 3.95**  **p < .001***  **M = 6.67**  **(3.20, 10.13)** |
| * p < .05; df= 45. Comparisons were calculated as row-labeled variable minus column-labeled variable. ‘M refers to the mean difference. 95% confidence intervals are listed. Among WJ-II subtest names, ‘C’ refers to those related to linguistic comprehension and ‘D’ refers to those related to decoding. Comparisons between contiguous scores when rank-ordered from lowest to highest are bolded (I.e., passage comprehension < sentence reading fluency < letter word identification < oral reading < word attack). | | | | |

|  | | | | | | |  |
| --- | --- | --- | --- | --- | --- | --- | --- |
| Table S4. Univariate analyses of WJ-IV subtest scores. | | | | | | |  |
| **Outcome: Child-Parent Behavioral Attunement** | | | | | | |  |
| **Variable** | **β**  **(SE)** | ***t*** | **p,**  **q** | **VIF** | **R^2^** | **AIC_c_** | **Power** |
| Oral Reading (C) | 0.11  (0.17) | 0.67 | 0.51, 0.51 | 1.00 | 0.07 | 131.86 | 0.09 |
| Passage Comprehension (C) | 0.20  (0.19) | 1.08 | 0.29, 0.41 | 1.00 | 0.08 | 131.09 | 0.19 |
| **Sentence Reading Fluency (C) ^a^** | **0.43**  **(0.18)** | **2.39** | **0.02*, 0.04*** | **1.00** | **0.17** | **126.50** | **0.75** |
| Word Attack (D) | -0.05  (0.17) | -0.30 | 0.77, 0.77 | 1.00 | 0.06 | 132.25 | 0.06 |
| Letter Word Identification (D) | 0.09  (0.16) | -0.92 | 0.56,  0.51 | 1.00 | 0.07 | 131.97 | 0.08 |
| ** p < .05; * q < .05.* Values reported come from regressions which included scores from a single WJ-II subtest and covariates as predictors.  Adjusted R^2^ values, standardized beta values (β) with standard errors (SE), t-values, variance inflation factors (VIF), second-order Akaike's information criterion (AICc), and power estimates are reported. Bolded rows reflect a statistically significant relationship. Both p values and q values which correct for false discovery rate are reported. Subtests labeled “C” are associated with linguistic comprehension, and those labeled with “D” are associated with phonemic decoding. | | | | | | | |

**^a^** This model also included a significant association between sex and behavioral attunement, such that female children received higher scores compared with male children (β = 0.74, p = .02).

| Table S5. Multivariate regression analyses for WJ-IV subtest scores. | | | | |  |
| --- | --- | --- | --- | --- | --- |
| **Outcome: Child-Parent Behavioral Attunement** | | | | |  |
| **Variable** | **β**  **(SE)** | ***t*** | ***p,***  ***q*** | **VIF** | **Power** |
| Intercept | 0.18  (0.20) | 0.86 | 0.39,  0.45 | - | - |
| Passage Comprehension (C) | 0.16  (0.20) | 0.76 | 0.45,  0.45 | 2.69 | 0.12 |
| **Sentence Reading Fluency (C)** | **0.54**  **(0.20)** | **2.75** | **0.01*,**  **0.04*** | **2.55** | **0.93** |
| **Word Attack (D)** | **-0.43**  **(0.17)** | **-2.51** | **0.02*,**  **0.05*** | **1.89** | **0.74** |
| **ASD Diagnosis** | **-0.98**  **(0.32)** | **-3.08** | **0.004*,**  **0.03*** | **1.55** | **0.84** |
| Sex | 0.56  (0.40) | 1.90 | 0.07,  0.12 | 1.23 | 0.30 |
| IQ | -0.01  (0.01) | -1.00 | 0.33,  0.45 | 2.13 | 0.15 |
| * p < .05; * q < .05. R^2^ = 0.32; AIC_c_ = 148.14. Results come from the full model including all WJ-IV subtest scores as well as covariates, with binary ASD diagnostic status in place of AQ-Child as a measure of ASD symptomatology.  Adjusted R^2^ values, standardized beta values (β) with standard errors (SE), t-values, variance inflation factors (VIF), second-order Akaike's information criterion (AICc), and power estimates are reported. Bolded rows reflect a statistically significant relationship. Both p values and q values which correct for false discovery rate are reported. ASD diagnosis was coded such that “0” refers to individuals with no diagnosis. The reference category for the sex variable was “male”. IQ refers to the child’s general conceptual ability score on the DAS-II assessment. Woodcock Johnson subtest age-standardized standard scores labeled “C” are associated with linguistic comprehension, and those labeled with “D” are associated with phonemic decoding. | | | | | |

| Table S6. Moderation analyses | | | |  |  |
| --- | --- | --- | --- | --- | --- |
| **Outcome: Child-Parent Behavioral Attunement** | | | |  |  |
| **a. AQ-Child** | **β**  ***(SE)*** | ***t*** | ***p,***  ***q*** | **VIF** | **Power** |
| Intercept | -0.10  (0.16) | -0.61 | 0.55,  0.55 | - | - |
| **AQ-Child** | **-0.36**  **(0.14)** | **-2.58** | **0.01*,**  **0.06** | **1.26** | **0.59** |
| **Sentence Reading Fluency Score (C)** | **0.35**  **(0.16)** | **2.15** | **0.04*,**  **0.08** | **1.74** | **0.57** |
| **AQ-Child * Sentence Reading Fluency Score (C)** | **0.28**  **(0.13)** | **2.19** | **0.03*,**  **0.08** | **1.05** | **0.37** |
| IQ | -0.13  (0.16) | -0.82 | 0.42,  0.50 | 1.72 | 0.24 |
| Sex | 0.47  (0.30) | 1.59 | 0.12,  0.18 | 1.27 | 0.11 |
| R^2^ | 0.32 |  |  |  |  |
| AIC_c_ | 121.04 |  |  |  |  |
| **Outcome: Child-Parent Behavioral Attunement** | | | | | |
| **b. AQ-Child** | **β**  ***(SE)*** | ***t*** | ***p,***  ***q*** | **VIF** | **Power** |
| Intercept | 7.21  (2.95) | 2.44 | 0.02,  0.12 | - | - |
| AQ-Child | -0.04  (0.04) | -1.01 | 0.32,  0.55 | 34.63 | 1.00 |
| Word Attack Score (D) | -0.02  (0.03) | -0.84 | 0.41,  0.55 | 7.71 | 0.39 |
| AQ-Child * Word Attack Score (D) | 1.8*10^-4^  (3.5*10^-4^) | 0.75 | 0.46,  0.55 | 1.13 | 0.08 |
| IQ | 0.02  (0.01) | 1.20 | 0.24,  0.55 | 1.35 | 0.15 |
| Sex | 0.31  (0.41) | 0.52 | 0.60,  0.60 | 31.66 | 0.60 |
| R^2^ | 0.16 |  |  |  |  |
| AIC_c_ | 155.81 |  |  |  |  |
| **Outcome: Child-Parent Behavioral Attunement** | | | |  |  |
| **c. ASD Diagnosis** | **β**  ***(SE)*** | ***t*** | ***p,***  ***q*** | **VIF** | ***Power*** |
| Intercept | 0.16  (0.21) | 0.77 | 0.44 | - | - |
| **ASD Diagnosis** | **-0.73**  **(0.30)** | **-2.40** | **0.02*,**  **0.13** | **1.37** | **0.56** |
| Sentence Reading Fluency Score (C) | 0.15  (0.21) | 0.72 | 0.47,  0.47 | 2.87 | 0.13 |
| **ASD Diagnosis * Sentence Reading Fluency (C)** | **0.52**  **(0.26)** | **2.02** | **0.05*,**  **0.15** | **2.21** | **0.63** |
| IQ | -0.18  (0.17) | -1.04 | 0.30,  0.46 | 1.88 | 0.16 |
| Sex | 0.46  (0.30) | 1.55 | 0.13,  0.26 | 1.25 | 0.22 |
| R^2^ | 0.30 |  |  |  |  |
| AIC_c_ | 122.12 |  |  |  |  |
| **Outcome: Child-Parent Behavioral Attunement** | | | | | |
| **d. ASD Diagnosis** | **β**  ***(SE)*** | ***t*** | ***p,***  ***q*** | **VIF** | ***Power*** |
| **Intercept** | **6.17***  **(1.86)** | **3.32** | **0.002*,**  **0.01** | **-** | - |
| ASD Diagnosis | -2.84  (2.22) | -1.28 | 0.21,  0.42 | 33.63 | 1.00 |
| Word Attack Score (D) | -0.02  (0.01) | -1.24 | 0.22,  0.42 | 2.37 | 0.27 |
| AQ-Child * Word Attack Score (D) | 0.02  (0.02) | 0.85 | 0.40,  0.42 | 1.12 | 0.09 |
| IQ | 0.01  (0.01) | 1.04 | 0.30,  0.42 | 1.45 | 0.13 |
| Sex | 0.35  (0.42) | 0.82 | 0.42,  0.42 | 30.34 | 0.96 |
| R^2^ | 0.14 |  |  |  |  |
| AIC_c_ | 156.89 |  |  |  |  |

** p < .05; * q < .05.* Adjusted R^2^ values, standardized beta values (β) with standard errors (SE), t-values, variance inflation factors (VIF), second-order Akaike's information criterion (AICc), and power estimates are reported. The reference category for the sex variable was “male”. IQ refers to the child’s general conceptual ability score on the DAS-II assessment. Woodcock Johnson (WJ-IV) subtest age-standardized standard scores labeled “C” are associated with linguistic comprehension.

Bolded rows reflect a statistically significant relationship. Both p values and q values which correct for false discovery rate are reported. Values reported reflect analyses of the moderating effect of (a-b) children’s scores on the autism spectrum quotient- child version (AQ-Child) and (c-d) diagnosis of ASD on the effects of behavioral attunement scores on skill in literacy subskills. The outcome variable is age-standardized sentence reading fluency score. Covariates include sex and IQ.

| Table S7. Robustness analysis evaluating whether results were equivalently driven by participants from both ASD diagnostic groups. | | | | | | |  |
| --- | --- | --- | --- | --- | --- | --- | --- |
| **a. Full Model**  **Outcome: Child-Parent Behavioral Attunement** | | | | | | |  |
| **i. NT Subgroup** | **β** | **β [*95% CI]*** | ***p, q*** | | ***P* [95% CI]** | |  |
| Intercept | 6.98*10^-4^ | [-0.38, 0.38] | 0.88, 0.88 | | [0.58, 0.99] | |  |
| Sentence Reading Fluency (C) | 0.47 | [-0.19, 1.13] | 0.16, 0.41 | | [0.09, 0.24] | |  |
| Word Attack (D) | -0.34 | [-0.94, 0.26] | 0.27, 0.41 | | [0.13, 0.44] | |  |
| AQ-Child | -0.11 | [-0.54, 0.31] | 0.59, 0.71 | | [0.40, 0.79] | |  |
| Sex | 0.26 | [-0.19, 0.71] | 0.26, 0.41 | | [0.10, 0.44] | |  |
| IQ | -0.33 | [-0.80, 0.13] | 0.16, 0.41 | | [0.05, 0.27] | |  |
| Residual M (SE) | -0.02 (0.20) | | | | | |  |
| MSE | 1.17 | | | | | |  |
| LOO-IC | 95.14 | | | | | |  |
| ELPD (SE) | -47.38 (5.38) | | | | | |  |
| **Outcome: Child-Parent Behavioral Attunement** | | | | | | |  |
| **ii. Autistic Subgroup** | **β** | **β [*95% CI]*** | ***p, q*** | | ***P* [95% CI]** | |  |
| Intercept | -9.05*10^-6^ | [-0.41, 0.41] | 0.82, 0.82 | | [0.51, 0.98] | |  |
| **Sentence Reading Fluency (C)** | **0.72** | **[0.12, 1.31]** | **0.03*, 0.18** | | **[5.5*10^-3^, 0.09]** | |  |
| Word Attack (D) | -0.21 | [-0.66, 0.25] | 0.30, 0.52 | | [0.12, 0.62] | |  |
| AQ-Child | -0.18 | [-0.62, 0.27] | 0.40, 0.52 | | [0.23, 0.62] | |  |
| Sex | 0.24 | [-0.24, 0.71] | 0.30, 0.52 | | [0.10, 0.53] | |  |
| IQ | 0.21 | [-0.35, 0.78] | 0.43, 0.52 | | [0.17, 0.63] | |  |
| Residual M (SE) | 1.4*10^-4^ (0.21) | | | |  | |  |
| MSE | 0.71 |  |  | |  | |  |
| LOO-IC | 47.37 |  |  | |  | |  |
| ELPD (SE) | -23.69 (4.00) | |  | |  | |  |
| **Outcome: Child-Parent Behavioral Attunement** | | | | | | |  |
| **iii. Full Sample** | **β** | **β [*95% CI]*** | ***p, q*** | | ***P* [95% CI]** | |  |
| **Intercept** | **5.46** | **[2.29, 8.63]** | **.001*, .006** | | **[7.1*10^-4^, .002]** | |  |
| **Sentence Reading Fluency (C)** | **0.04** | **[0.01, 0.06]** | **.007*, .02** | | **[.002, .012]** | |  |
| **Word Attack (D)** | **-0.02** | **[-0.04, -3.1*10^-4^]** | **.05*, .09** | | **[.021, .087]** | |  |
| **AQ-Child** | **-0.02** | **[-0.03, -4.7*10^-3^]** | **.01*, .12** | | **[.004, .017]** | |  |
| Sex | 0.70 | [-0.12, 1.52] | .10, .12 | | [.035, .176] | |  |
| IQ | -3.42*10^-3^ | [-0.03, 0.02] | .79, .79 | | [.537, .989] | |  |
| Residual M (SE) | -0.03 (0.18) | | | | | |  |
| MSE | 1.40 |  |  | |  | |  |
| LOO-IC | 151.57 |  |  | |  | |  |
| ELPD (SE) | -75.78 (6.71) | |  | |  | |  |
| **b. Interaction Model**  **Outcome: Child-Parent Behavioral Attunement** | | | | | | |  |
| **i. NT Subgroup** | **β** | **β [*95% CI]*** | ***p, q*** | | ***P* [95% CI]** | |  |
| Intercept | 0.02 | [-0.37, 0.41] | 0.87, 0.87 | | [0.47, 0.99] | |  |
| AQ-Child | -0.16 | [-0.58, 0.27] | 0.46, 0.68 | | [0.62, -0.16] | |  |
| Sentence Reading Fluency (C) | 0.28 | [-0.27, 0.83] | 0.31, 0.68 | | [0.17, 0.52] | |  |
| AQ-Child * Sentence Reading Fluency (C) | 0.18 | [-0.46, 0.81] | 0.57, 0.68 | | [0.90, 0.18] | |  |
| Sex | 0.21 | [-0.24, 0.66] | 0.36, 0.68 | | [0.56, 0.21] | |  |
| IQ | -0.39 | [-0.85, 0.08] | 0.10, 0.60 | | [0.18, 0.56] | |  |
| Residual M, SE | -7.9*10^-3^ (0.20) | | | | | |  |
| MSE | 1.15 | | | | | |  |
| LOO-IC | 95.17 | | | | | |  |
| ELPD (SE) | -47.58 (5.39) | | | | | |  |
| **Outcome: Child-Parent Behavioral Attunement** | | | | | | |  |
| **ii. Autistic Subgroup** | **β** | **β [*95% CI]*** | ***p, q*** | | ***P* [95% CI]** | |  |
| Intercept | 8.1*10^-3^ | [-0.42, 0.44] | 0.82, 0.82 | | [0.532, 0.986] | |  |
| AQ-Child | -0.19 | [-0.69, 0.32] | 0.43, 0.65 | | [0.269, 0.661] | |  |
| Sentence Reading Fluency (C) | 0.64 | [0.02, 1.27] | 0.06, 0.34 | | [0.013, 0.173] | |  |
| AQ-Child * Sentence Reading Fluency (C) | -0.06 | [-0.80, 0.69] | 0.82, 0.82 | | [0.508, 0.947] | |  |
| Sex | 0.25 | [-0.26, 0.77] | 0.30, 0.65 | | [0.125, 0.514] | |  |
| IQ | 0.23 | [-0.38, 0.85] | 0.43, 0.65 | | [0.164, 0.789] | |  |
| Residual M, SE | -0.15 (0.22) | | | | | |  |
| MSE | 0.81 | | | | | |  |
| LOO-IC | 47.84 | | | | | |  |
| ELPD (SE) | -23.92 (4.00) | | | | | |  |
| **Outcome: Child-Parent Behavioral Attunement** | | | | | | |  |
| **iii. Full Sample** | **β** | **β [*95% CI]*** | | ***p, q*** | | ***P* [95% CI]** | |
| **Intercept** | **9.44** | **[3.86, 15.02]** | | **1.7*10^-3^, 0.01** | | **[6.3*10^-4^, 3.2*10^-3^]** | |
| **AQ-Child** | **-0.08** | **[-0.15, -0.02]** | | **0.01*, 0.04** | | **[.007, .034]** | |
| Sentence Reading Fluency (C) | -0.02 | [-0.07, 0.03] | | 0.45, 0.45 | | [.315, .745] | |
| **AQ-Child * Sentence Reading Fluency (C)** | **6.7*10^-4^** | **[4.1*10^-5^, 1.3*10^-3^]** | | **0.04*, 0.08** | | **[.021, .081]** | |
| Sex | 0.63 | [-0.18, 1.44] | | 0.13, 0.20 | | [.077, .234] | |
| IQ | -0.01 | [-0.04, 0.02] | | 0.43, 0.45 | | [.240, .630] | |
| Residual M, SE | -0.02 (0.18) | |  | |  | |  |
| MSE | 1.39 |  |  | |  | |  |
| LOO-IC | 150.78 |  |  | |  | |  |
| ELPD (SE) | -75.39 (6.71) | |  | |  | |  |
| ** p < .05; * q < .05.* Re-estimation of the full model (a) and significant interaction model (b) using leave-one-out cross-validation (LOO-CV) for the (i) neurotypical (NT) subgroup, (ii) autistic subgroup, and (iii) full sample. Standardized beta values (β) and their 95% confidence intervals (CI), p values and q values which correct for false discovery rate, and 95% CIs for p-values are reported. Per model, means (M) and standard errors (SE) of the residuals for model iterations, mean squared error (MSE), leave-one-out information criterion (LOO-IC), and estimated Expected Log Predictive Density (ELPDS) with its standard error are reported to reflect relative model fit. NT refers to neurotypical participants. Bolded rows reflect statistically significant relationships.  The reference category for the sex variable was “male”. IQ refers to the child’s general conceptual ability score on the DAS-II assessment. Woodcock Johnson (WJ) subtest age-standardized standard scores labeled “C” are associated with linguistic comprehension, and those labeled with “D” are associated with phonemic decoding. | | | | | | |  |

The goal of this analysis was to determine whether the same pattern of effects emerged when analyzing data independently for neurotypical (NT; *Table A6.a-bi*) or autism spectrum disorder (ASD; *Table A6.b*) child participant groups. This would indicate that neither group singularly drove significant results from the full sample. To achieve this, we separately re-estimated the multivariate regression models from our primary analysis for the autistic (N = 16) and neurotypical (NT) participant subgroups (N = 29), as well as for the full sample, using leave-one-out cross-validation (LOO-CV; Wong, 2015; Yates et al., 2023). This method provided robust results within the smaller sub-samples and allowed for direct comparisons with the full sample.

For each LOO-CV iteration, models were trained on all data points except one, which was used for testing. We extracted standardized regression coefficients, their 95% confidence intervals, p-values, and residuals from each iteration and averaged these estimates. We calculated the log-likelihood for each test observation, using the normal distribution with mean predictions and standard deviation from model residuals. The LOO information criterion (LOO-IC) was determined as -2 times the sum of log-likelihoods across all test observations.

We assessed the model's predictive performance using the expected log predictive density (ELPD), computed as the total log-likelihood, with its standard error approximated by the square root of the number of observations. Additionally, we calculated the mean squared error (MSE) and the mean and standard error (SE) of residuals across iterations. All models controlled for child sex and IQ as covariates. For a discussion of the results, see the main manuscript discussion section: *“Robustness analysis of participant subgroups based on ASD diagnosis.”* Potential shortcomings of this modeling approach are addressed in the limitations.
